# Identification of a novel six‐gene signature with potential prognostic and therapeutic value in cervical cancer

**DOI:** 10.1002/cam4.4054

**Published:** 2021-09-08

**Authors:** Xinyu Qu, Zhiwen Shi, Jingjing Guo, Chenyan Guo, Junjun Qiu, Keqin Hua

**Affiliations:** ^1^ Department of Gynaecology, Obstetrics and Gynaecology Hospital Fudan University Shanghai China; ^2^ Shanghai Key Laboratory of Female Reproductive Endocrine‐Related Diseases Shanghai China

**Keywords:** cervical cancer, gene signature, prognosis, tumour immune microenvironment

## Abstract

**Introduction:**

Cervical cancer has high mortality, high recurrence and poor prognosis. Although prognostic biomarkers such as clinicopathological features have been proposed, their accuracy and precision are far from satisfactory. Therefore, novel biomarkers are urgently needed for disease surveillance, prognosis prediction and treatment selection.

**Materials:**

Differentially expressed genes (DEGs) between cervical cancer and normal tissues from three microarray datasets extracted from the Gene Expression Omnibus platform were identified and screened. Based on these DEGs, a six‐gene prognostic signature was constructed using cervical squamous cell carcinoma and endocervical adenocarcinoma data from The Cancer Genome Atlas. Next, the molecular functions and related pathways of the six genes were investigated through gene set enrichment analysis and co‐expression analysis. Additionally, immunophenoscore analysis and the QuartataWeb Server were employed to explore the therapeutic value of the six‐gene signature.

**Results:**

We discovered 178 overlapping DEGs in three microarray datasets and established a six‐gene (APOC1, GLTP, ISG20, SPP1, SLC24A3 and UPP1) prognostic signature with stable and excellent performance in predicting overall survival in different subgroups. Intriguingly, the six‐gene signature was closely associated with the immune response and tumour immune microenvironment. The six‐gene signature might be used for predicting response to immune checkpoint inhibitors (ICIs) and the six genes may serve as new drug targets for cervical cancer.

**Conclusion:**

Our study established a novel six‐gene (APOC1, GLTP, ISG20, SPP1, SLC24A3 and UPP1) signature that was closely associated with the immune response and tumour immune microenvironment. The six‐gene signature was indicative of aggressive features of cervical cancer and therefore might serve as a promising biomarker for predicting not only overall survival but also ICI treatment effectiveness. Moreover, three genes (UPP1, ISG20 and GLTP) within the six‐gene signature have the potential to become novel drug targets.

## Graphical abstract

Our study established a novel six‐gene (APOC1, GLTP, ISG20, SPP1, SLC24A3 and UPP1) signature that was closely associated with the immune response and the tumour immune microenvironment. The six‐gene signature was indicative of aggressive features of cervical cancer and therefore might serve as a promising biomarker for predicting not only overall survival but also immune checkpoint inhibitor treatment effectiveness. Moreover, three genes (UPP1, ISG20 and GLTP) within the six‐gene signature have the potential to become novel drug targets.

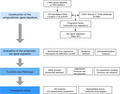

## 1 INTRODUCTION

Cervical cancer ranks fourth in both incidence and mortality in women worldwide, with approximately 500,000 new cases and 250,000 deaths occurring annually.^1^ Despite continuous advancements in treatment, including surgery, radiotherapy, chemotherapy and targeted or immunotherapy,^2^, ^3^, ^4^ the 5‐year overall survival for cervical cancer is reported to be approximately 50%~65%,^5^, ^6^ with a high recurrence rate and poor prognosis.^7^ It is known that clinicopathological features, such as International Federation of Gynecology and Obstetrics stage, lymph node metastasis, lymphovascular space invasion (LVSI), deep stromal infiltration and parametrial involvement, are traditional prognostic factors. However, these characteristics are not precise enough or temporally dynamic,^8^ and they can only be obtained postoperatively.

With the rapid development of molecular biology techniques, cancer biomarkers for prognosis prediction are increasingly emerging. In cervical cancer, although squamous cell carcinoma antigen (SCCA)^9^ has been applied in clinical practice, its specificity and sensitivity are far from satisfactory.^10^ For example, SCCA also increases in other types of squamous cell carcinoma^11^, ^12^, ^13^ and even in nontumour diseases.^14^ In addition, it is not good for identifying and monitoring cervical adenocarcinoma. Therefore, the identification of novel biomarkers and a deeper understanding of the molecular mechanisms of cervical cancer are essential for disease surveillance, prognosis prediction and treatment selection.

High‐throughput microarray and RNA sequencing (RNA‐seq) technologies integrated with bioinformatics analyses have emerged as promising tools for detecting genetic alterations in the processes of tumour formation, recurrence and metastasis. Gene Expression Omnibus (GEO, https://www.ncbi.nlm.nih.gov/geo/),^15^ a large database of gene chip data maintained by the National Center of Biotechnology and The Cancer Genome Atlas (TCGA, https://portal.gdc.cancer.gov/)^16^ offering comprehensive cancer genomic expression, clinicopathological and survival information, are two commonly used public databases. In‐depth exploration of such databases assists researchers in screening, identifying and validating cancer biomarkers, providing strong support for diagnosis, prognostication and individualised treatment selection for different types of tumours; for example, SETD7 was identified as a diagnostic biomarker for colorectal cancer,^17^ TFAP2B was identified as a prognostic predictor for endometrial cancer^18^ and phospho‐STAT1 was identified as a biomarker for patient selection for immunotherapy in breast cancer.^19^ However, to the best of our knowledge, previous studies on prognostic biomarkers for cervical cancer have been either based on individual microarray dataset or database,^20^, ^21^ thus failed to present convincing results, or failed to identify the function and clinical value of the studied genes.^22^ Therefore, more comprehensive research uniting different data sources and delving further into gene function in cervical cancer is urgently needed.

In the current study, to explore potential prognostic biomarkers for cervical cancer, we first analysed three mRNA microarray datasets from the GEO platform and obtained differentially expressed genes (DEGs) between cervical cancer tissues and normal tissues. Subsequently, DEGs with prognostic value were identified with the data from TCGA. Notably, we established **a novel six‐gene (APOC1, GLTP, ISG20, SPP1, SLC24A3 and UPP1) prognostic signature** and discovered for the first time that this signature was associated not only with intrinsic aggressive features but also with the tumour immune microenvironment. Furthermore, we also investigated the therapeutic value of the six‐gene signature and found that it could help predict the response to immune checkpoint inhibitors (ICIs) and that three genes (GLTP, ISG20 and UPP1) in the six‐gene signature might serve as potential drug targets for cervical cancer.

## 2 MATERIALS AND METHODS

### 2.1 Data collection and preprocessing

Microarray datasets (GSE7803, GSE9750, GSE138080 and GSE127265) were obtained from GEO (https://www.ncbi.nlm.nih.gov/geo/).^15^ Detailed information is summarised in Table S1. Gene probes were converted into corresponding gene symbols according to the annotation information on the platform. The Robust Multi‐array Average method was used to normalise and log2‐transform data, which were prepared for the next‐step screening to identify DEGs. The cervical squamous cell carcinoma and endocervical adenocarcinoma (CESC) dataset of the TCGA database were downloaded from Xena (https://xena.ucsc.edu/) including normalised gene expression RNA‐seq fragments per kilobase of exon model per million mapped fragments (FPKM) data, clinicopathological data and survival data.

### 2.2 DEG screening in GEO

The ‘Limma’ package of R software (version 3.6.2) was applied to identify DEGs in three microarray datasets (GSE7803, GSE9750 and GSE138080) on the GEO platform with thresholds of |log_2_ fold change| >1 and adjusted p value <0.05. Three volcano plots demonstrating the DEGs and a Venn diagram indicating overlapping genes were generated with R software.

### 2.3 Construction and evaluation of the six‐gene prognostic signature

We first applied univariate Cox regression analysis to identify DEGs with prognostic value in the TCGA CESC dataset. Then, a multivariate Cox regression analysis was conducted with the ‘step Akaike information criterion (AIC)’ method. Genes with statistical significance (*p* < 0.05) were selected to establish a multiple‐gene prognostic signature. The risk score predicting overall survival was calculated as follows: ∑coefficient∗geneexpression. To assess the accuracy of the prognostic signature, time‐dependent receiver operating characteristic (ROC) curves predicting survival at different time points were drawn and the area under the curve (AUC) value was calculated in R software using the ‘survival ROC’ package. Additionally, the patients were divided into a high‐risk group and a low‐risk group according to the median risk score. Kaplan‐Meier curves were generated and the log‐rank test was performed between these two groups with the ‘survival’ package of R software. To examine whether the six‐gene signature we constructed was an independent risk factor, we performed univariate and multivariate Cox regression analyses employing both the six‐gene signature and clinicopathological features. A nomogram was used to visualise the multivariate Cox model and calibration curves were generated to test the concordance of the prediction model at 1, 3, 5 and 10 years. In addition, to evaluate the robustness of the six‐gene prognostic signature, we performed an extra subgroup analysis and the results were presented as a forest plot. The nomogram, calibration curves and forest plot were generated with R software with the packages ‘survival’, ‘rms’ and ‘forestplot’, respectively.

### 2.4 Gene set enrichment analysis

Gene set enrichment analysis (GSEA) is a computational method that determines whether a defined set of genes exhibit statistical and concordant differences between two phenotypes. We used GSEA software^23^, ^24^ (version 4.0.3) to explore the enrichment of hallmark gene sets in patients with high‐risk and low‐risk according to the six‐gene signature. Furthermore, pathways related to high expression and low expression patterns of the six studied genes were also investigated individually.

### 2.5 Analysis of the correlations between the prognostic genes and tumour‐infiltrating immune cells

RNA‐seq data were transformed into abundances of immune cells via CIBERSORT^25^ in R software. We analysed the correlation between the six‐gene signature risk score as well as single gene expression values and the abundances of immune cells. Scatter plots were generated in R software when a correlation (*R* > 0.3 and *p* < 0.001) was detected. Associations between gene expression and important immune markers of a variety of immune cells, including monocytes, tumour‐associated macrophages, M1 macrophages, M2 macrophages, neutrophils, natural killer cells, dendritic cells (DCs), B cells, T cells (general), CD8^+^ T cells, T helper 1 (Th1) cells, T helper 2 cells, follicular helper T cells, T helper 17 cells, regulatory T cells and exhausted T cells, were investigated with TIMER,^26^, ^27^ a web tool providing gene co‐expression results adjusted by tumour purity. A heatmap was then generated with GraphPad Prism (version 8.2.1) with colours representing Spearman's correlation coefficient (*R*) and ‘*’ representing the *p* value. The absolute value of the correlation coefficient (*R*) indicated the strength of the correlation: 0–0.3, weak; 0.3–0.5, moderate; 0.5–0.7 strong; and 0.7–1, very strong.

### 2.6 Immunophenoscore analysis

The immunophenoscore (IPS) was derived with machine learning algorithms from the representative gene expression in four major categories of factors related to immunogenicity, including effector cells, immunosuppressive cells, major histocompatibility complex (MHC) molecules and immunomodulators. The IPS was calculated based on the *z*‐scores of gene expression using a scale ranging from 0 to 10. It has been validated that higher IPSs are associated with stronger immunogenicity, indicating a better response to ICIs.^28^ The IPSs of CESC patients were acquired from The Cancer Immunome Atlas (https://tcia.at/home).

### 2.7 Searching the QuartataWeb Server for potential drugs

The QuartataWeb Server^29^ (http://quartata.csb.pitt.edu.) is a user‐friendly website designed for polypharmacological and chemogenomics analyses, whose original data sources are DrugBank^30^ and STITCH.^31^ Users can conveniently obtain access to information on experimentally verified protein‐drug/chemical interactions and computationally predicted interactions. With the help of the QuartataWeb Server, we identified the potential drugs targeting the proteins encoded by the genes we were interested in.

### 2.8 Statistical analysis

Continuous variables are described as the mean ± SE or the median, and categorical variables are presented as the frequency (*n*) and proportion (%). Differences in the variables between groups were tested using *t* tests, nonparametric tests, chi‐square tests, or ANOVA tests. All hypothetical tests were two‐sided and a *p* value less than 0.05 was considered statistically significant. Statistical analyses were performed with R software (version 3.6.2), SPSS (version 25) and GraphPad Prism (version 8.2.1).

## 3 RESULTS

### 3.1 A total of 178 overlapping DEGs were identified from 3 GEO datasets of cervical cancer

**FIGURE 1:**
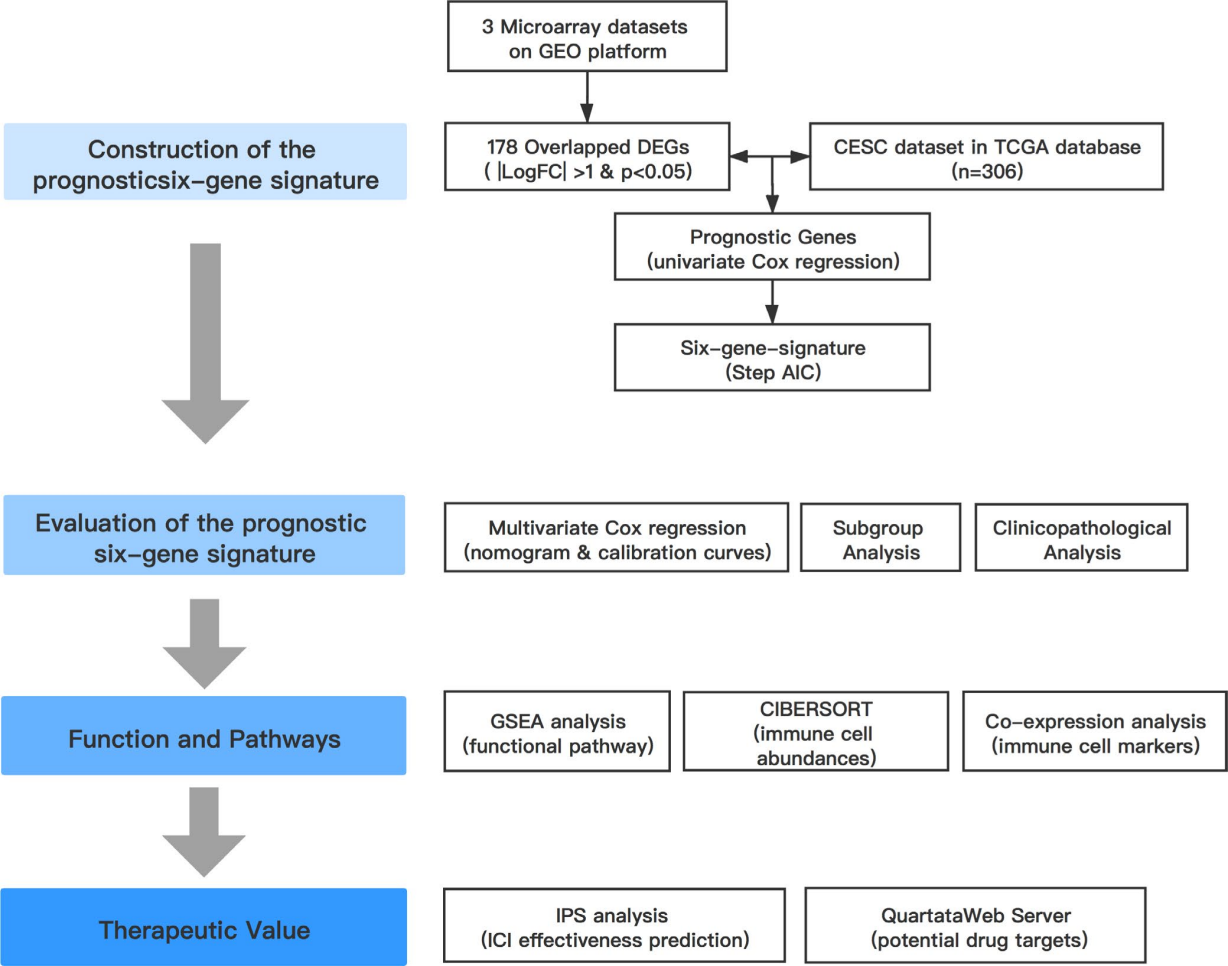
Flow chart of the current study. AIC, Akaike information criterion; CESC, Cervical squamous cell carcinoma and endocervical adenocarcinoma; DEG, differentially expressed genes; GEO, Gene Expression Omnibus; GSEA, Gene set enrichment analysis; ICI, immune checkpoint inhibitors; IPS, immunophenoscore; TCGA, the Cancer Genome Atlas

**FIGURE 2:**
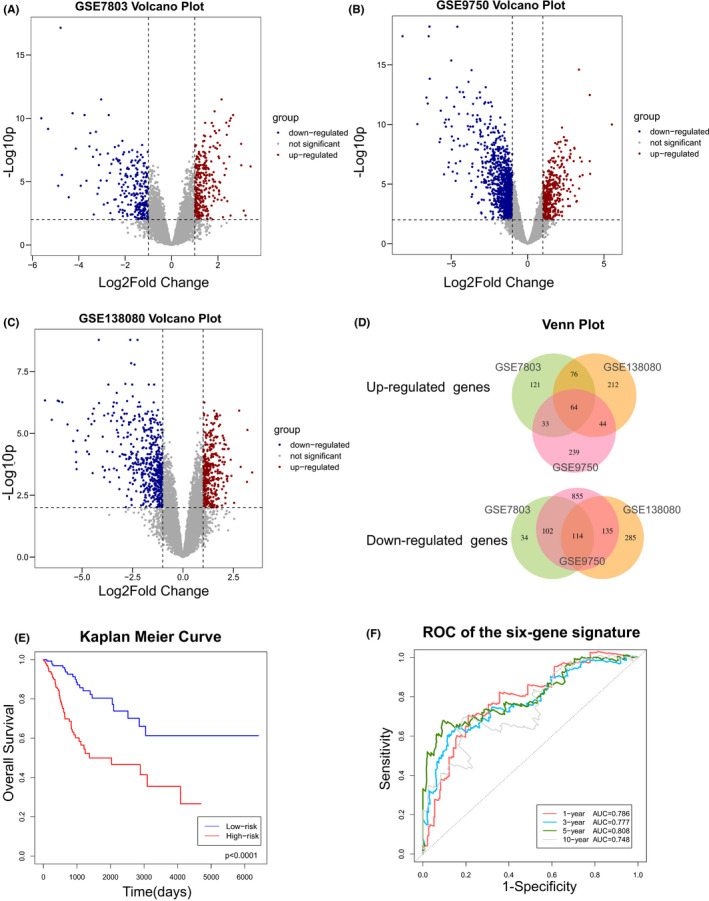
(A–C) Volcano plot of the differentially expressed genes (DEGs) discovered in (A) GSE7803 dataset, (B) GSE9750 dataset and (C) GSE138080 dataset. (D) One hundred and seventy‐eight overlapped genes within the above three GEO datasets. (E) Kaplan–Meier curves of the six‐gene signature low‐risk group and high‐risk group with significant statistical difference demonstrating that a higher risk score led to a worse prognosis. (F) Receiver operating characteristic (ROC) curve of the six‐gene signature predicting OS at 1, 3, 5 and 10 years. GEO, Gene Expression Omnibus

The whole study was conducted according to the flow chart (Figure 1). We analysed 3 cervical cancer datasets (GSE7803, GSE9750 and GSE138080) on the GEO platform, identifying 543 (293 upregulated and 250 downregulated), 1602 (396 upregulated and 1206 downregulated) and 914 (380 upregulated and 534 downregulated) DEGs, respectively. Notably, 178 DEGs overlapped amongst the three datasets, including 64 upregulated genes and 114 downregulated genes (Figure 2A–D). Whether these DEGs play significant roles in the development of cervical cancer and offer great prognostic value were the next questions we sought to answer.

### 3.2 **A promising six‐gene prognostic signature was constructed with TCGA data**.

To explore prognostic biomarkers, we performed survival analysis using the CESC dataset from the TCGA database, which includes 306 cervical cancer samples with RNA‐seq data, survival data and clinicopathological data (Table 1). First, we analysed the 178 DEGs by univariate Cox regression analysis and found 36 DEGs with potential prognostic value. Then, multivariate Cox regression analysis was performed with the ‘step AIC’ method. Of note, we found that six genes (APOC1, GLTP, ISG20, SPP1, SLC24A3 and UPP1) had remarkable statistical significance (*p* < 0.05) which were subsequently included in the overall survival prediction model (Table 2). Notably, we established a six‐gene signature that could predict overall survival for the first time and the risk score was calculated as follows: 0.3322*UPP1‐0.6835*GLTP + 0.2347*SLC24A3+0.2777*SPP1‐0.4078*ISG20‐0.3020*APOC1 (concordance = 0.764, SE = 0.028). Additionally, we divided 306 patients into a high‐risk group and a low‐risk group using the median risk score as the cut‐off value. Kaplan–Meier curves and log‐rank tests were employed to compare the overall survival between high‐risk and low‐risk patients (Figure 2E). The results showed that a higher risk score predicted a shorter overall survival. Time‐dependent ROC curves for the six‐gene signature were generated (Figure 2F) and the AUC values for the 1‐, 3‐, 5‐and 10‐year survival predictions were 0.786, 0.777, 0.808 and 0.748, respectively. Collectively, these results suggested that the novel six‐gene (APOC1, GLTP, ISG20, SPP1, SLC24A3 and UPP1) panel we constructed might serve as a promising prognostic biomarker.

**TABLE 1:** Clinicopathological features of CESC dataset in TCGA database

Characteristics	Entire cohort *N* = 306
Age (years)	
≤46	156 (51.0%)
>46	150 (49.0%)
Histology	
SCC	254 (83.0%)
AC	47 (15.4%)
ASC	5 (1.6%)
Clinical stage	
I	162 (52.9%)
II	69 (22.5%)
III	46 (15.0%)
IV	22 (7.2%)
NA	7 (2.3%)
Grade	
G1	18 (5.9%)
G2	135 (44.1%)
G3	118 (38.6%)
G4	1 (0.3%)
NA	34 (11.1%)
Lymph node	
N0	134 (43.8%)
N1	61 (19.9%)
NA	111 (36.3%)
LVSI	
Absent	71 (23.2%)
Present	81 (26.5%)
NA	154 (50.3%)
Metastasis	
M0	116 (37.9%)
M1	11 (3.6%)
NA	179 (58.5%)
Radiation	
Yes	144 (47.1%)
No	56 (18.3%)
NA	106 (34.6%)

Abbreviations: CESC, cervical squamous cell carcinoma and endocervical adenocarcinoma; LVSI, lymphovascular space invasion; NA, not available; TCGA, The Cancer Genome Atlas.

**TABLE 2:** Hazard ratios of each gene in the six‐gene signature

Gene symbol	Official full name	HR	95% CI	*p* value
APOC1	Apolipoprotein C1	0.739	0.615–0.889	0.001
GLTP	Glycolipid transfer protein	0.505	0.385–0.662	<0.001
ISG20	Interferon stimulated exonuclease gene 20	0.665	0.495–0.895	0.007
SLC24A3	Solute carrier family 24 member 3	1.265	1.038–1.541	0.020
SPP1	Secreted phosphoprotein 1	1.320	1.140–1.528	<0.001
UPP1	Uridine phosphorylase 1	1.394	1.124–1.729	0.003

### 3.3 **The six‐gene signature was an independent prognostic factor**.

**FIGURE 3:**
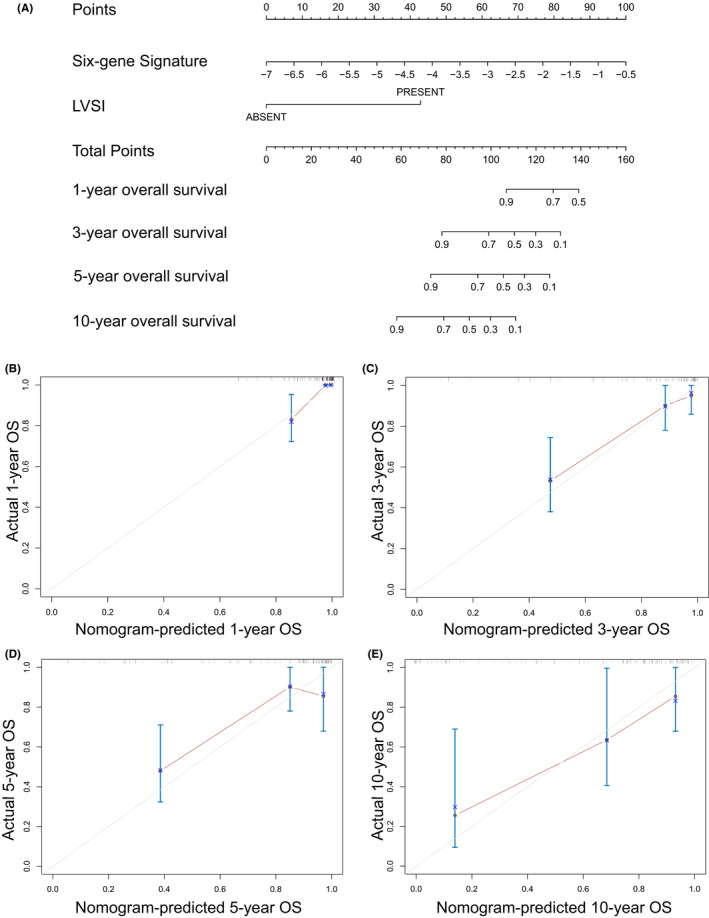
(A) Nomogram combining the six‐gene signature and LVSI predicting OS for 1, 3, 5 and 10 years. (B–E) Calibration curves of the multivariate Cox model predicting OS (D) at 1 year, (E) at 3 years, (F) at 5 years and (G) at 10 years. LVSI, lymphovascular space invasion

**FIGURE 4:**
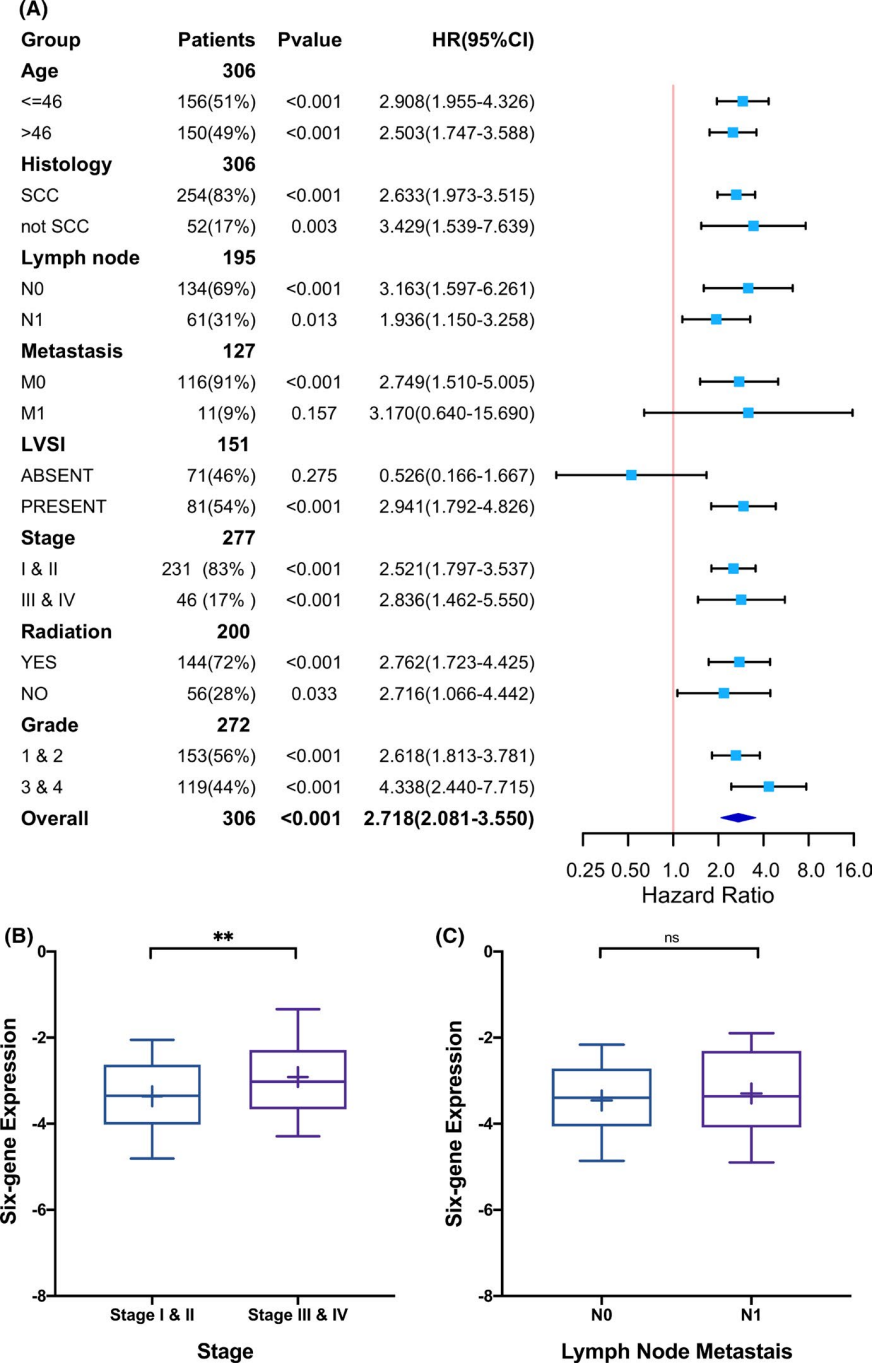
(A) Forest Plot illustrating the hazard ratios of the six‐gene signature for overall survival in different clinicopathological subgroups. (B) Box plot of the six‐gene expression between earlier clinical stage and advanced clinical stage. (C) Box plot of the six‐gene expression between LNM‐N0 group and LNM‐N1 group

To further examine whether the six‐gene signature was an independent prognostic factor, we performed not only univariate but also multivariate Cox regression analysis (Table 3). Clinical stage (stage III and IV vs. stage I and II), lymph node status (N1 vs. N0), lymphovascular infiltration (present vs. absent), metastasis (M1 vs. M0) and the six‐gene signature proved to be prognostic factors according to the univariate model, while the multivariate model included LVSI and the six‐gene signature (concordance = 0.86, SE = 0.033), demonstrating that the six‐gene signature could serve as an independent prognostic factor. Then a nomogram was constructed based on LVSI and the six‐gene signature to predict the overall survival of cervical cancer patients and the calibration curves predicting 1, 3, 5, 10‐year overall survival (OS) were illustrated (Figure 3). To evaluate the stability and reliability of the six‐gene signature, we conducted a subgroup analysis (Figure 4A). A higher risk score predicted poorer overall survival in most subgroups, with a p value less than 0.05. Overall, we proved that the six‐gene panel can be an independent prognostic biomarker.

**TABLE 3:** Univariate and multivariate Cox model predicting overall survival

Characteristics	Patients	Univariate Cox model			Multivariate Cox model		
		HR	95% CI	*p* value	HR	95% CI	*p* value
Age	306	1.016	0.999–1.034	0.062			
Histology	306						
SCC/AC and ASC		1.027	0.539–1.955	0.936			
Clinical stage	299						
III and IV/I and II		2.369	1.457–3.854	**<0.001**			
Grade	272						
3 and 4/1 and 2		0.8894	0.527–1.502	0.661			
Lymph node	195						
N1/N0		2.844	1.446–5.593	**0.002**			
LVSI	152						
Present/Absent		10.460	2.466–44.360	**0.001**	12.228	2.875–51.9995	**0.0007**
Metastasis	127						
M1/M0		3.555	1.187–10.640	**0.023**			
Radiation	200						
No/Yes		0.918	0.415–2.032	0.833			
Six‐gene signature	306	2.718	2.081–3.550	**<0.001**	2.454	1.561–3.858	**0.0001**

Abbreviation: LVSI, lymphovascular space invasion.

### 3.4 **The six‐gene signature we established was related to aggressive features**.

To further analyse whether the six‐gene signature was related to clinicopathological features, we conducted the statistical analysis of the differences between the six‐gene signature low‐risk group and high‐risk group (Table S2). We noticed that a high‐risk score was linked with lymph node metastasis (*p* = 0.022) and showed a trend for being related to the advanced clinical stage (*p* = 0.076). In addition, we compared the risk scores in different subgroups (Figure 4B,C). Discrepancies existed between the early‐stage group and advanced‐stage group (*p* = 0.005), further confirming the association between the six‐gene panel and clinical stage of cervical cancer. But risk scores had no differences between the N1 group and N0 group (*p* = 0.3253), indicating that the correlation between the six‐gene signature and lymph node metastasis was not robust and convincing enough. The statistical difference in Table S2 (*p* = 0.022) was probably due to the missing data. Additionally, by analysing another cervical cancer dataset on the GEO platform (GSE 127265), the association between the six‐gene signature risk score and more advanced clinical stage was confirmed (Figure S1).

In a word, a high six‐gene signature risk score was linked to the advanced clinical stage representing the aggressive features of cervical cancer. Therefore, we wondered how these six genes enact their ability to promote tumour aggressiveness.

### 3.5 **The six‐gene signature was found to be closely associated with immune‐related pathways**.

**FIGURE 5:**
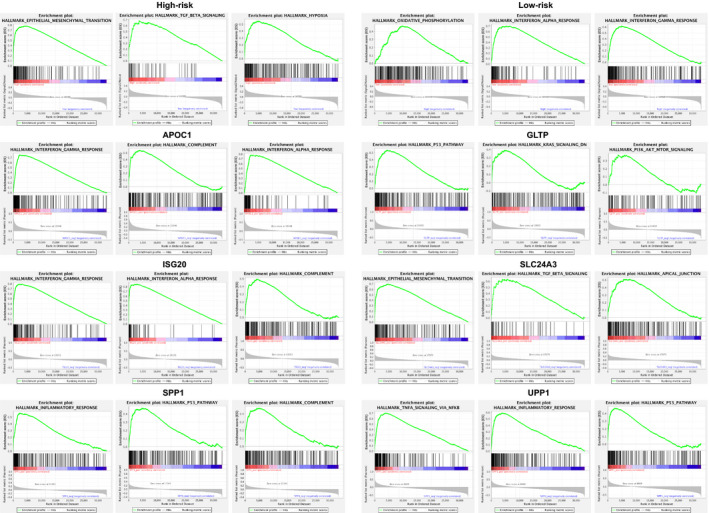
GSEA Enrichment plots indicating the top three related pathways of the six‐gene prognostic signature and the six studied genes including APOC1, GLTP, ISG20, SLC24A3, SPP1 and UPP1. GSEA, gene set enrichment analysis

Subsequently, we deeply examined the gene functions and pathways related to the signature with GSEA (Figure 5). We found that epithelial‐mesenchymal transition, TGF‐beta pathway and hypoxia were enriched in the high‐risk group, while oxidative phosphorylation, interferon‐alpha response and interferon‐gamma response were enriched in the low‐risk group. These results indicated that the poor prognosis of the high‐risk score group was related to the activation of common carcinogenesis pathways; in contrast, the better prognosis of the low‐risk score group was linked to a positive immune response. Additionally, we also studied the functional pathways correlated with the six genes individually. Interestingly, APOC1, ISG20, SPP1 and UPP1 were found to be closely connected with the immune response and inflammation‐related pathways. Collectively, these results implied that the six genes might regulate the immune microenvironment or mediate immune reactions, thus influencing tumour aggressiveness and disease prognosis.

### 3.6 **The six‐gene signature was correlated with the tumour immune microenvironment**.

**FIGURE 6:**
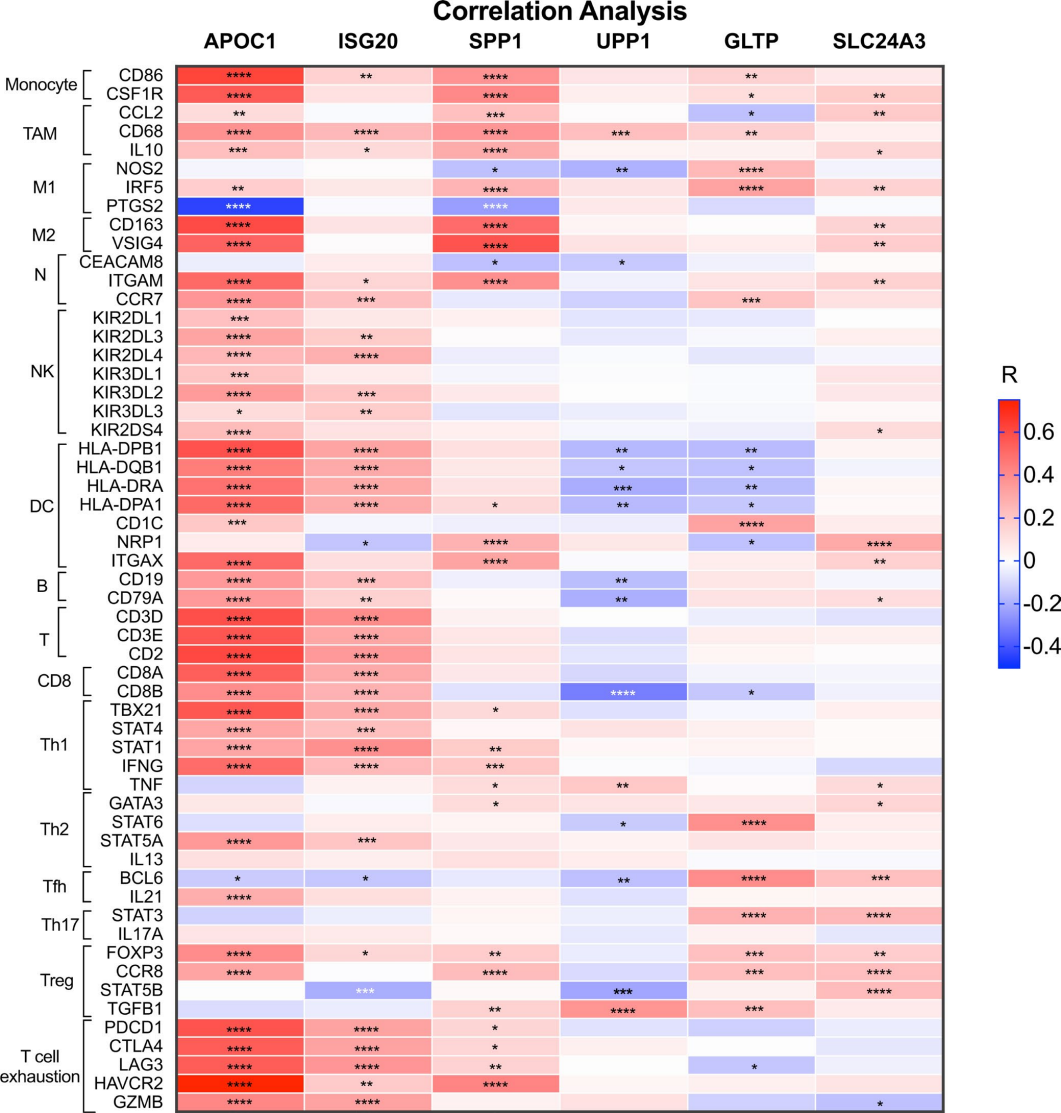
Heatmap of the six prognostic genes expression correlated with immune markers colours representing the correlation coefficients (*R*) and ‘*’standing for *p* value. ****p < 0.0001; ***p < 0.001; **p < 0.01; *p < 0.05. The correlations were adjusted by tumour purity

To further confirm whether the six‐gene signature was indicative of the immune microenvironment, we explored the association between the six‐gene signature and tumour immune microenvironment in terms of various tumour‐infiltrating immune cells (Table 4) through CIBERSORT and correlation analysis. We observed that the six‐gene risk score was positively correlated with the abundances of resting CD4^+^ memory T cells, M0 macrophages and activated mast cells but negatively associated with the abundances of CD8^+^ T cells and resting mast cells. We also investigated the correlations between the six genes and the abundances of immune cells individually (Figure S2). Notably, we found that APOC1 had strong links with M2 macrophages, activated DCs, CD4^+^ resting memory T cells and CD8^+^ T cells. To further test whether the six genes potentially regulated immune cells, we conducted co‐expression analysis between the six genes and immune markers demonstrating the state and function of immune cells (Figure 6). We discovered that APOC1 had a strong correlation with immune markers pf monocytes, M2 macrophages, DCs and T cells, especially exhausted T cells. Of note, APOC1 had the strongest association (*R* = 0.744) with HAVCR2 (also named TIM‐3), a marker of exhausted T cells. Additionally, ISG20 exhibited a similar expression pattern as APOC1, while SPP1 was positively associated with M2 macrophages. Overall, we confirmed that the novel six‐gene signature we established was closely associated with the tumour immune microenvironment.

**TABLE 4:** Correlations between the six‐gene signature and 22 types of immune cells

Immune cell	Correlation	*p* Value	Significance
B cells naive	−0.162	0.005	**
B cells memory	−0.037	0.523	
Plasma cells	−0.118	0.043	*
T cells CD8	−**0.352**	**0.000**	***
T cells CD4 naive	NA	NA	NA
T cells CD4 memory resting	**0.343**	**0.000**	***
T cells CD4 memory activated	−0.239	**0.000**	***
T cells follicular helper	−0.098	0.094	
T cells regulatory (Tregs)	−0.158	0.006	**
T cells gamma delta	−0.062	0.289	
NK cells resting	0.106	0.070	
NK cells activated	−0.052	0.371	
Monocytes	0.068	0.244	
Macrophages M0	**0.346**	**0.000**	***
Macrophages M1	−0.100	0.089	
Macrophages M2	−0.032	0.581	
Dendritic cells resting	−0.097	0.095	
Dendritic cells activated	0.023	0.688	
Mast cells resting	−**0.328**	**0.000**	***
Mast cells activated	**0.396**	**0.000**	***
Eosinophils	0.072	0.215	
Neutrophils	0.228	**0.000**	***

* *p* < 0.05

** *p* < 0.01

*** *p* < 0.001.

The absolute value of the correlation was more than 0.3 and *p* value was less than 0.001 in the rows with bold texts implying a relatively robust relation with statistical significance (in bold).

### 3.7 The six‐gene signature exhibited potential therapeutic value

**FIGURE 7:**
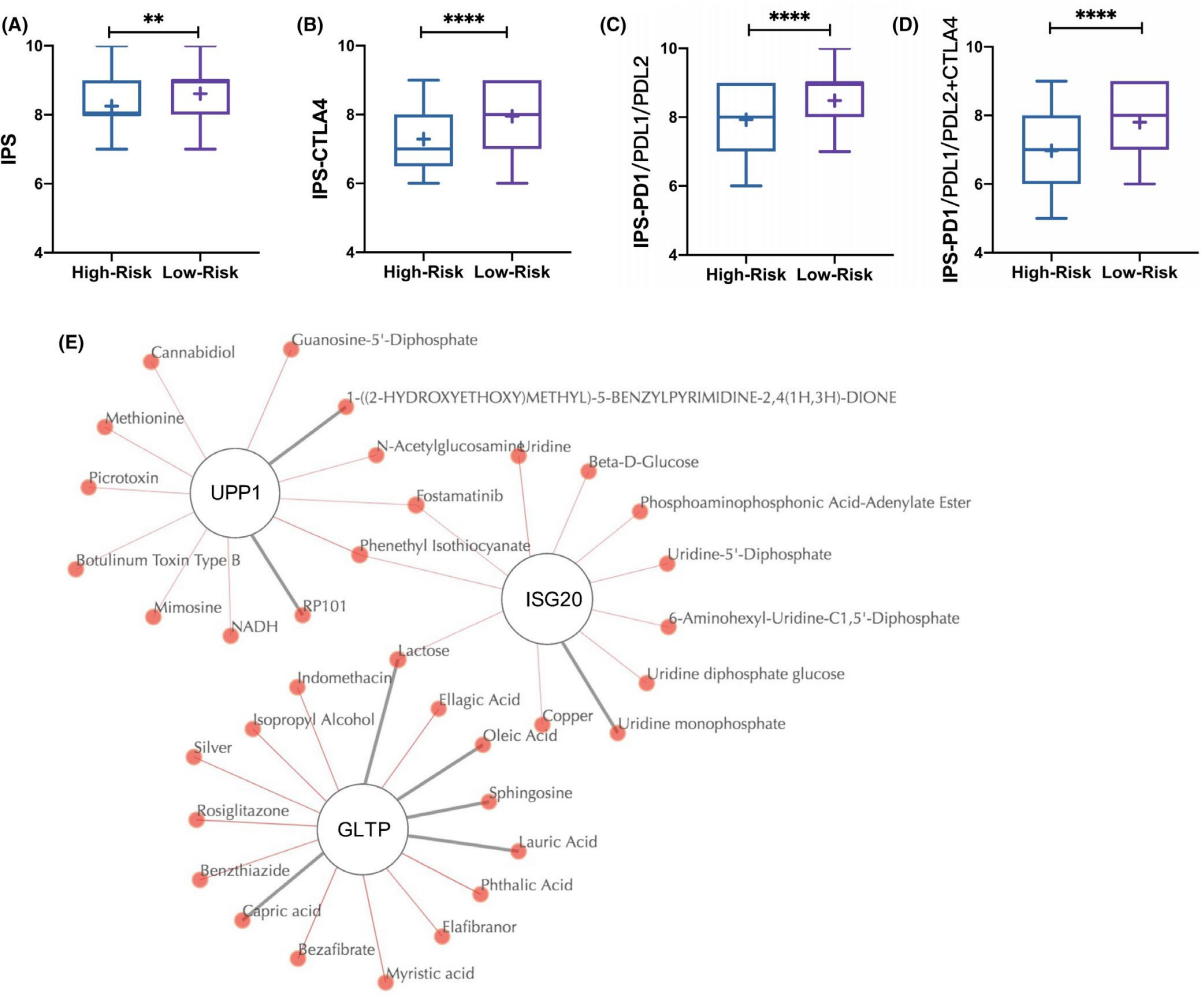
(A) IPS, (B) IPS‐CTLA4, (C) IPS‐PD1/PDL1/PDL2 and (D) IPS‐PD1 PD1/PDL1/PDL2+CTLA4 in the six‐gene signature high‐risk group and low‐risk group. (E) The known (grey thick lines) and predicted (red thin lines) drugs targeting UPP1, ISG20 and GLTP. IPS, immunophenoscore

We sought to determine whether the six‐gene signature could provide therapeutic value. First, we used the IPS to predict ICI effectiveness within patients classified as high‐risk and low‐risk according to the six‐gene signature (Figure 7A–D). The low‐risk group exhibited higher IPSs, as well as higher IPS‐CTLA4 and IPS‐PD1/PDL1/PDL2+CTLA4 scores, than the high‐risk group, representing higher immunogenicity, thus predicting a better response to ICIs including CTLA4 blockade and PDL1 blockade. In summary, the six‐gene signature might assist in predicting ICI treatment effectiveness. Second, we searched the QuartataWeb Server for potential drugs targeting the proteins encoded by the six prognostic genes. We identified 1, 5 and 2 known small molecule drugs targeting ISG20, GLTP and UPP1, respectively (Table S3). Notably, fostamatinib and phenethyl isothiocyanate targeted both UPP1 and ISG20, and lactose interacted with ISG20 and GLTP (Figure 7E). Overall, the six‐gene signature exhibited an appreciable therapeutic value in predicting ICI treatment effectiveness and highlighting potential drug targets (GLTP, UPP1 and ISG20).

## 4 DISCUSSION

Despite the rapid development of molecular biology and sequencing technologies, previous studies exploring novel biomarkers^32^, ^33^, ^34^ in cervical cancer were mostly based on a single microarray dataset or database with limited validity^20^, ^21^ or lacked a clear description of molecular function.^22^ In the present study, by comprehensively integrating three GEO datasets and CESC data from the TCGA database, we established a novel six‐gene (APOC1, ISG20, SPP1, UPP1, GLTP and SLC24A3) prognostic signature. The six‐gene signature was related to the clinical stage and proved to be an independent prognostic factor for cervical cancer with stable performance in different subgroups. Subsequently, a strong link between tumour immune environment and the six genes had been discovered. Intriguingly, the six‐gene risk score was positively correlated with the abundances of resting CD4^+^ T cells and activated mast cells but negatively correlated with the abundances of CD8^+^ T cells, activated CD4^+^ T cells and resting mast cells. That is, a lower risk score represented higher abundances of CD8^+^ T cells and activated CD4^+^ T cells, which have been widely reported to be effector cells^35^, ^36^, ^37^, ^38^ in the tumour microenvironment generally leading to a better prognosis. In addition, significant associations between vessel density and mast cell abundance have been found in cervical carcinoma,^39^, ^40^ providing an explanation for the link between high six‐gene risk score and poor prognosis: high activated mast cell abundance and increased angiogenesis. To determine the therapeutic value of the six‐gene signature, we performed IPS analysis and concluded that a lower six‐gene risk score predicted a better response to ICIs than a higher six‐gene risk score. Additionally, we identified drugs targeting GLTP, UPP1 and ISG20, offering new directions for drug development.

Gene functions and mechanisms are of vital importance in determining clinical value and applications. Hence, we placed much emphasis on investigating how the six genes from our signature function and what pathways they mediate (Table S4). In the field of cervical cancer, a previous study found that SPP1 was upregulated in cancer tissues compared with normal tissues.^41^ Experiments proved that inhibiting SPP1 could inhibit proliferation, induce apoptosis and improve the chemosensitivity of cervical cancer cells. In addition, ISG20 was also reported to be differentially expressed in cervical cancer tissues.^42^ However, the other four genes within the signature have rarely been studied in cervical cancer. In our current study, through GSEA, we found that protective genes for overall survival, especially APOC1 and ISG20, had positive correlations with immune‐related pathways, including the interferon‐alpha pathway, interferon‐gamma pathway and complement pathway, implying that an effective immune response might lead to good prognosis. Moreover, the risk genes predicting poor survival, particularly SPP1 and UPP1, were linked with common carcinogenesis pathways as well as immune‐related pathways such as the inflammatory response and TNF signalling via NFKB. In summary, our study discovered that the six prognostic genes, especially APOC1, ISG20, SPP1 and UPP1, had the potential to regulate immune response thus contributing to cervical cancer aggressiveness. These findings greatly expanded our understanding of the function of these genes in cervical cancer.

We also studied whether the expression of the six prognostic genes reflected the immune microenvironment or immune response. Through co‐expression analyses, we verified that APOC1, ISG20 and SPP1 had notably strong correlations with tumour‐infiltrating immune cells, highlighting new directions for further research. (1) **APOC1**, as a protective factor for overall survival, was found to be closely related to the abundances of multiple tumour‐infiltrating immune cells, such as M2 macrophages, activated DCs and CD8^+^ T cells. Correspondingly, APOC1 had strong associations with 18 immune markers. Of note, APOC1 had an extremely strong relationship with HAVCR2 (also named TIM‐3), a marker of exhausted T cells. In addition, APOC1 was positively correlated with the abundance of M2 macrophages but negatively correlated with PTGS2, a marker of M1 macrophages, indicating a potential association with macrophage polarisation, which is critical in the antitumour response.^43^, ^44^ Hence, we suggested that APOC1 might affect tumour malignancy and cancer prognosis by regulating T cell exhaustion and macrophage polarisation. (2) Additionally, we observed that **ISG20**, as a protective gene for survival, was correlated with T cells and DCs. As ISG20 participates in the antiviral response, we hypothesised that ISG20 might participate in the anti‐HPV immune response by activating T cells or DCs and thus influence the overall survival of cervical cancer patients. (3) Moreover, we discovered that **SPP1** expression was positively correlated with the abundance of M2 macrophages as well as the expression of corresponding immune markers (CD163 and VSIG4). M2 macrophages exert anti‐inflammatory and pro‐tumour effects, promoting the progression and metastasis of a variety of tumours, such as breast cancer and gastric cancer.^45^, ^46^ Therefore, we hypothesised that SPP1 might contribute to tumour aggressiveness and influence cervical cancer prognosis by regulating M2 macrophages, but this hypothesis requires further investigation and validation.

The present study had several limitations. First, extra clinical cohorts with large‐scale samples were not recruited to accomplish validation of the newly constructed six‐gene signature. Second, the value of the six‐gene signature in immunotherapy prediction was not shown in clinical cohorts. Finally, experiments to verify the hypothesis we proposed regarding the function and mechanisms of the six prognostic genes, as well as the predicted drug‐protein interactions, were not performed yet. In the future, clinical validation and experiments would be conducted to verify our current conclusions.

In conclusion, our current study represented the first attempt to construct a novel six‐gene (APOC1, GLTP, ISG20, SPP1, SLC24A3 and UPP1) signature that comprehensively integrated GEO and TCGA data. With a deeper investigation of the gene functions and pathways, we found that the six‐gene signature was closely associated with the immune response and tumour immune microenvironment. The six‐gene signature was indicative of aggressive features of cervical cancer and could serve as a promising biomarker for predicting not only overall survival but also ICI treatment effectiveness. Moreover, three genes (UPP1, ISG20 and GLTP) within the signature have the potential to become novel drug targets.

## CONFLICT OF INTEREST

The authors report no conflict of interest.

## AUTHOR CONTRIBUTION

Conceptualisation: Xinyu Qu, Junjun Qiu; Methodology: Xinyu Qu, Zhiwen Shi, Jingjing Guo; Data acquisition: Jingjing Guo, Chenyan Guo; Writing‐original draft: Xinyu Qu; Writing‐review & editing: all authors; Supervision: Keqin Hua, Junjun Qiu.

## Supporting information

Fig S1

Fig S2

Table S1‐S4

Figlegends

Reference

## Data Availability

Four microarray datasets (GSE7803, GSE9750, GSE138080, GSE127265) were obtained, respectively, from: https://www.ncbi.nlm.nih.gov/geo/query/acc.cgi?acc=GSE7803; https://www.ncbi.nlm.nih.gov/geo/query/acc.cgi?acc=GSE9750; https://www.ncbi.nlm.nih.gov/geo/query/acc.cgi?acc=GSE138080 and https://www.ncbi.nlm.nih.gov/geo/query/acc.cgi?acc=GSE127265. The CESC dataset of the TCGA database was downloaded from Xena (https://xena.ucsc.edu/). The IPSs of cervical patients of the TCGA database were acquired from The Cancer Immunome Atlas (https://tcia.at/home).
