# Supplementary figures and images for: Identification of a novel six‐gene signature with potential prognostic and therapeutic value in cervical cancer
Source: Cancer Med. 2021 Sep 8;10(19):6881–96. doi: 10.1002/cam4.4054 (PMC8495282; doi:10.1002/cam4.4054)

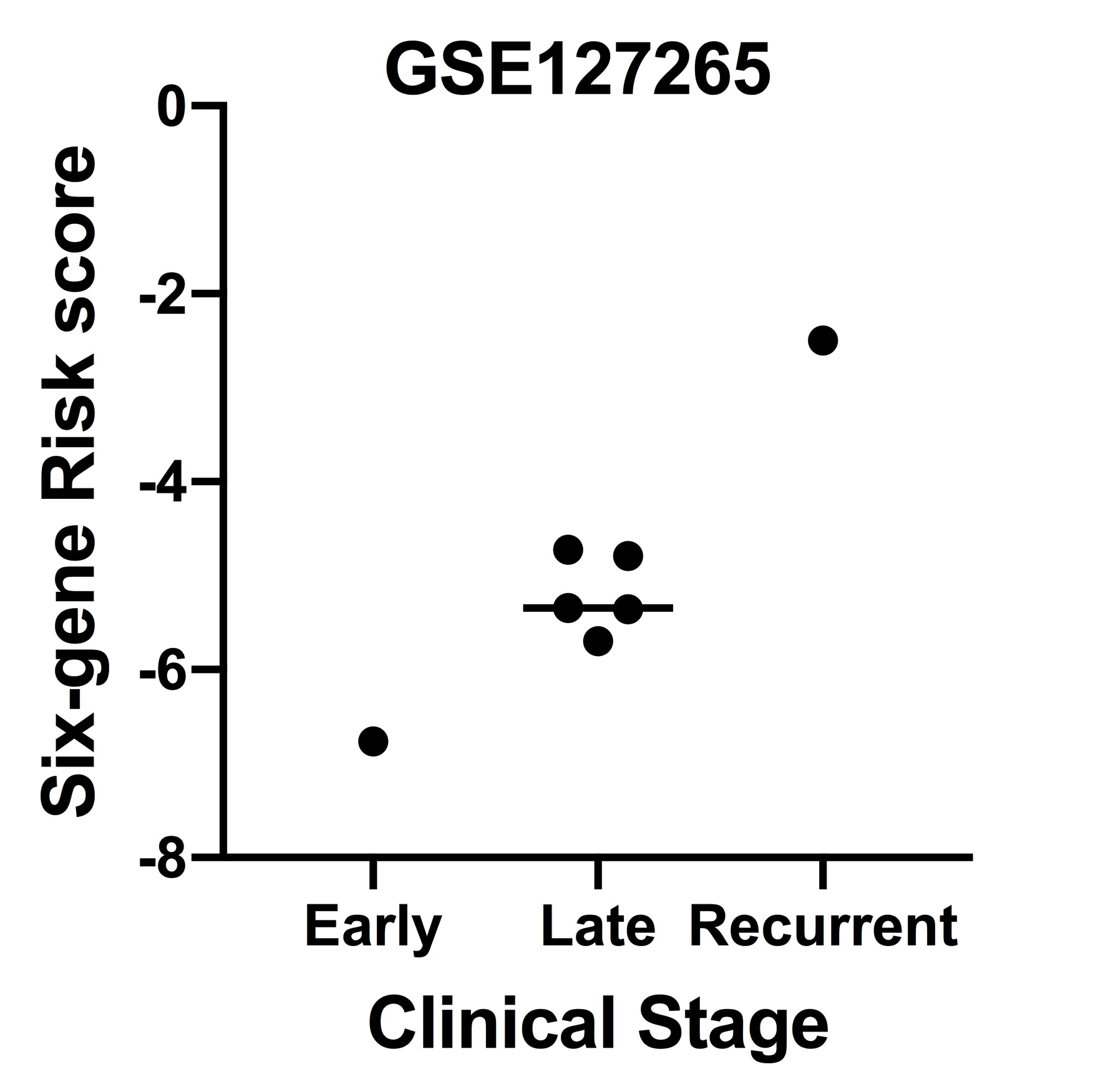

Supplement: Supplementary file 1 — Fig S1 [file CAM4-10-6881-s005.jpg]

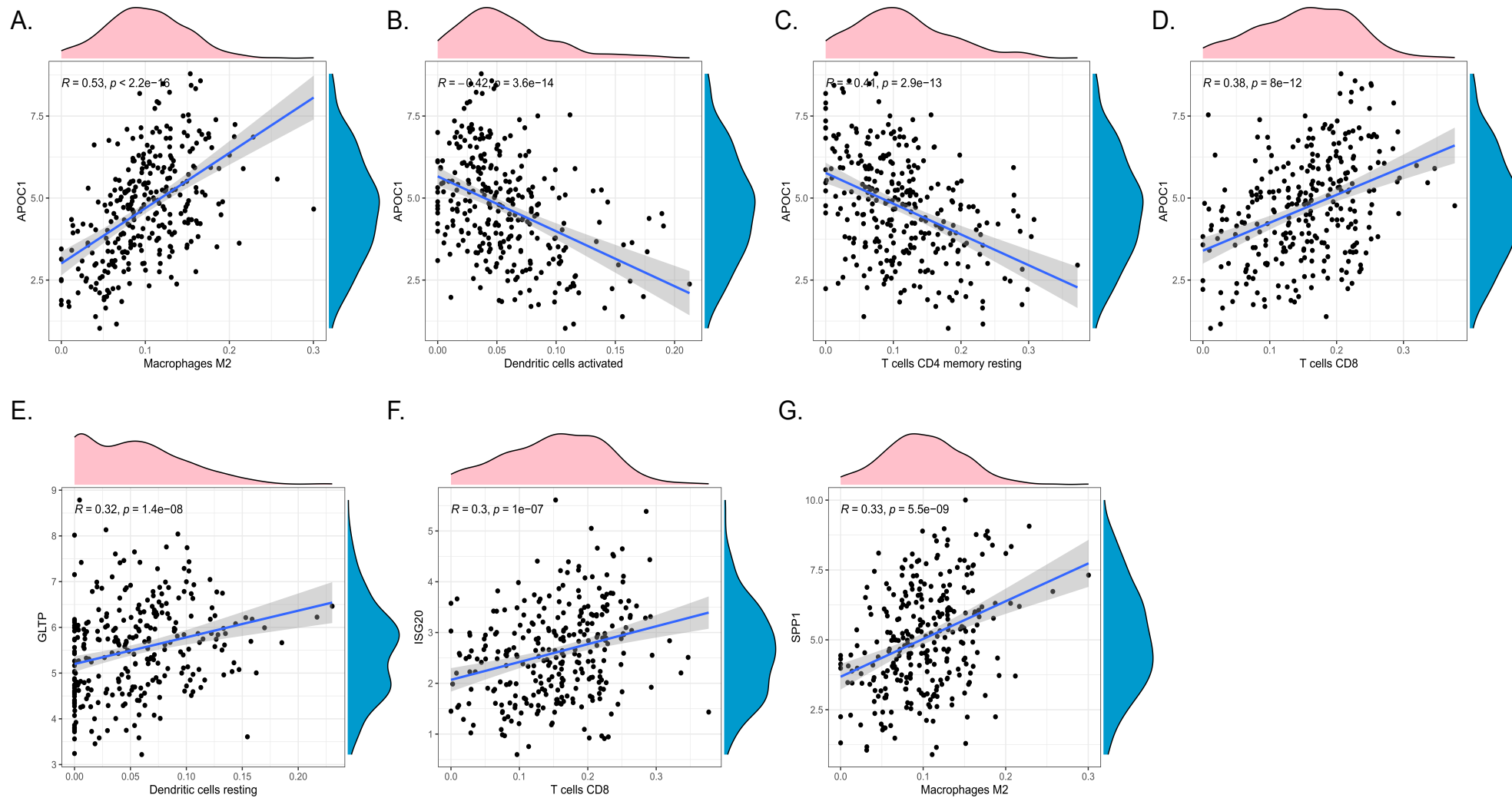

Supplement: Supplementary file 2 — Fig S2 [file CAM4-10-6881-s003.pdf]
