# Supplementary material for: Identification of a novel six‐gene signature with potential prognostic and therapeutic value in cervical cancer
Source: Cancer Med. 2021 Sep 8;10(19):6881–96. doi: 10.1002/cam4.4054 (PMC8495282; doi:10.1002/cam4.4054)
Supplement: Supplementary file 3 — Table S1‐S4 [file CAM4-10-6881-s002.docx]

**Supplements**

**Supplementary Table S1 Basic information of the 4 GEO datasets**

|  | **Cervical Cancer VS Normal tissue** | **Platform** |
| --- | --- | --- |
| GSE7803 | 21(SCC)VS 10 | GPL96 Affymetrix Human Genome U133A Array |
| GSE9750 | 33(28SCC&5AC) 21 | GPL96 Affymetrix Human Genome U133A Array |
| GSE138080 | 10(SCC)VS 10 | GPL4133 Agilent-014850 Whole Human Genome Microarray |
| GSE127265 | 7 (SCC)  Stage IB:1,  Stage IIB-IV:5, Recurrent:1 | GPL23126 Affymetrix Human Clariom D Assay |

**Supplementary Table S2 Clinicopathological features between the Low-risk group and the High-risk group**

| **Clinicopathological Feature** | | **Low-Risk Group** | **High-Risk Group** | **P Value** |
| --- | --- | --- | --- | --- |
| Age | | 47.9 (14.4) | 48.4(13.3) | 0.790 |
| Histology | SCC | 129 (84.3%) | 125(81.7%) | 0.543 |
|  | Not SCC | 24 (15.7%) | 28 (18.3%) |  |
| Clinical Stage | I & II | 124(81.0%) | 107(69.9%) | **0.076** |
|  | III & IV | 26(17.0%) | 42(27.5%) |  |
|  | NA | 3 (2.0%) | 4(2.6%) |  |
| Grade | 1&2 | 79(51.6%) | 74(48.4%) | 0.837 |
|  | 3&4 | 58(37.9%) | 61(39.9%) |  |
|  | NA | 16(10.5%) | 18(11.8%) |  |
| Lymph Node | N0 | 76(49.7%) | 58(37.9%) | **0.022** |
|  | N1 | 33(21.6%) | 28(18.3%) |  |
|  | NA | 44(28.8%) | 67(43.8%) |  |
| LVSI | Absent | 37(24.2%) | 34(22.2%) | 0.886 |
|  | Present | 41(26.8%) | 40(26.1%) |  |
|  | NA | 75(49.0%) | 79(51.6%) |  |
| Metastasis | M0 | 64(41.8%) | 52(34.0%) | 0.285 |
|  | M1 | 4(2.6%) | 7(4.6%) |  |
|  | NA | 85(55.6%) | 94(61.4%) |  |
| Radiation | No | 30(19.6%) | 26(17.0%) | 0.243 |
|  | Yes | 77(50.3%) | 67(43.8%) |  |
|  | NA | 46(30.1%) | 60(39.2%) |  |

**Supplementary Table S3 The known drugs interacting with UPP1, ISG20 and GLTP**

| **Targeted gene** | **Type** | **Drug ID** | **Drug** | **Drug structure** | **Group** |
| --- | --- | --- | --- | --- | --- |
| UPP1 | Small Molecular Drug | DB07437 | 1-((2-HYDROXYETHOXY)METHYL)-5-BENZYLPYRIMIDINE-2,4(1H,3H)-DIONE | 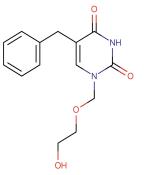 | Experimental |
| UPP1 | Small Molecular Drug | DB05041 | RP101 | No structure available | Investigational |
| GLTP | Small Molecular Drug | DB04465 | Lactose | 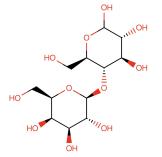 | Approved;  Investigational |
| GLTP | Small Molecular Drug | DB03203 | Sphingosine | 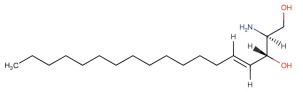 | Experimental |
| GLTP | Small Molecular Drug | DB03017 | Lauric Acid | 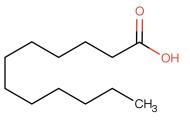 | Approved;  Experimental |
| GLTP | Small Molecular Drug | DB03600 | Capric acid | 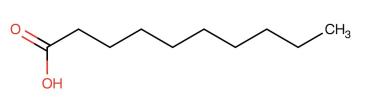 | Experimental |
| GLTP | Small Molecular Drug | DB04224 | Oleic Acid | 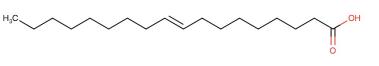 | Approved;  Investigational |
| ISG20 | Small Molecular Drug | DB03685 | Uridine monophosphate | 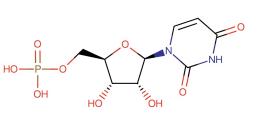 | Experimental |

**Supplementary Table S4 Biological function and previous studies of the Six genes**

| Gene | Biological function | Previous studies in oncology |
| --- | --- | --- |
| **APOC1** | Encodes apolipoprotein C1 playing a central role in both lipoprotein metabolism and monocyte differentiation | Diagnostic and prognostic value in gastric carcinoma ^[1]^, colorectal carcinoma ^[2]^ and lung cancer ^[3]^ |
| **GLTP** | Encodes glycolipid transfer protein closely related to lipid metabolism | Induce necroptosis of colon carcinoma cells and inhibit tumour cell proliferation ^[4]^ |
| **ISG20** | Stimulated by interferon and plays a critical role in the antiviral immune response ^[5-6]^ | Overexpressed in oral cancer ^[7]^ and hepatocellular carcinoma ^[8]^ |
| **SPP1** | Involved in the attachment of osteoclasts to the mineralized bone matrix and acts as a cytokine upregulating interferon-gamma and interleukin-12 | Reported to be a biomarker of colorectal cancer ^[9]^, gastric cancer ^[10]^ and hepatocellular carcinoma ^[11]^ |
| **UPP1** | Functions in the degradation and salvage of pyrimidine ribonucleosides | Overexpressed in various tumour tissues compared with normal tissues, including breast cancer and head and neck cancer tissues ^[12]^; reported to predict survival in brain glioma ^[13]^ |
| **SLC24A3** | Involved in intracellular calcium homeostasis and electrical conduction | Few reports in oncology |
