# Supplementary material for: Identification of a novel six‐gene signature with potential prognostic and therapeutic value in cervical cancer
Source: Cancer Med. 2021 Sep 8;10(19):6881–96. doi: 10.1002/cam4.4054 (PMC8495282; doi:10.1002/cam4.4054)
Supplement: Supplementary file 4 — Figlegends [file CAM4-10-6881-s001.docx]

**Figure S1.** The six-gene signature risk score of the samples in GSE 127265.

**Figure S2.** A. Scatter plot exhibiting the positive correlation between APOC1 expression and the abundances of M2 macrophages. B. Scatter plot exhibiting the negative correlation between APOC1 expression and the abundances of activated dendritic cells. C. Scatter plot exhibiting the negative correlation between APOC1 expression and the CD4 resting memory T cells. D. Scatter plot exhibiting the positive correlation between APOC1 expression and the abundances of CD8 T cells. E. Scatter plot exhibiting the positive correlation between GLTP expression and the abundances of resting dendritic cells. F. Scatter plot exhibiting the positive correlation between ISG20 expression and the abundances of CD8 T cells. G. Scatter plot exhibiting the positive correlation between SPP1 expression and the abundances of M2 macrophages.
