# Supplementary material for: Identification of a novel six‐gene signature with potential prognostic and therapeutic value in cervical cancer
Source: Cancer Med. 2021 Sep 8;10(19):6881–96. doi: 10.1002/cam4.4054 (PMC8495282; doi:10.1002/cam4.4054)
Supplement: Supplementary file 5 — Reference [file CAM4-10-6881-s004.docx]

1. Yi J, Ren L, Wu J, Li W, Zheng X, Du G, Wang J. Apolipoprotein C1 (APOC1) as a novel diagnostic and prognostic biomarker for gastric cancer. Ann Transl Med. 2019 Aug;7(16):380. doi: 10.21037/atm.2019.07.59. PMID: 31555694; PMCID: PMC6736826.

2. Ren H, Chen Z, Yang L, Xiong W, Yang H, Xu K, Zhai E, Ding L, He Y, Song X. Apolipoprotein C1 (APOC1) promotes tumor progression via MAPK signaling pathways in colorectal cancer. Cancer Manag Res. 2019 May 29;11:4917-4930. doi: 10.2147/CMAR.S192529. PMID: 31213910; PMCID: PMC6549782.

3. Ko HL, Wang YS, Fong WL, Chi MS, Chi KH, Kao SJ. Apolipoprotein C1 (APOC1) as a novel diagnostic and prognostic biomarker for lung cancer: A marker phase I trial. Thorac Cancer. 2014 Nov;5(6):500-8. doi: 10.1111/1759-7714.12117. Epub 2014 Oct 23. PMID: 26767044; PMCID: PMC4704334.

4. Mishra SK, Stephenson DJ, Chalfant CE, Brown RE. Upregulation of human glycolipid transfer protein (GLTP) induces necroptosis in colon carcinoma cells. Biochim Biophys Acta Mol Cell Biol Lipids. 2019 Feb;1864(2):158-167. doi: 10.1016/j.bbalip.2018.11.002. Epub 2018 Nov 22. PMID: 30472325; PMCID: PMC6448591.

5. Wu N, Nguyen XN, Wang L, Appourchaux R, Zhang C, Panthu B, Gruffat H, Journo C, Alais S, Qin J, Zhang N, Tartour K, Catez F, Mahieux R, Ohlmann T, Liu M, Du B, Cimarelli A. The interferon stimulated gene 20 protein (ISG20) is an innate defense antiviral factor that discriminates self versus non-self translation. PLoS Pathog. 2019 Oct 10;15(10):e1008093. doi: 10.1371/journal.ppat.1008093. PMID: 31600344; PMCID: PMC6805002.

6. Park YK, Lee SY, Lee AR, Kim KC, Kim K, Kim KH, Choi BS. Antiviral activity of interferon-stimulated gene 20, as a putative repressor binding to hepatitis B virus enhancer II and core promoter. J Gastroenterol Hepatol. 2020 Aug;35(8):1426-1436. doi: 10.1111/jgh.14986. Epub 2020 Feb 9. PMID: 31951295; PMCID: PMC7497004.

7. Miyashita H, Fukumoto M, Kuwahara Y, Takahashi T, Fukumoto M. ISG20 is overexpressed in clinically relevant radioresistant oral cancer cells. Int J Clin Exp Pathol. 2020 Jul 1;13(7):1633-1639. PMID: 32782682; PMCID: PMC7414473.

8. Lin SL, Wu SM, Chung IH, Lin YH, Chen CY, Chi HC, Lin TK, Yeh CT, Lin KH. Stimulation of Interferon-Stimulated Gene 20 by Thyroid Hormone Enhances Angiogenesis in Liver Cancer. Neoplasia. 2018 Jan;20(1):57-68. doi: 10.1016/j.neo.2017.10.007. Epub 2017 Nov 29. PMID: 29195126; PMCID: PMC5721268.

9.Xu C, Sun L, Jiang C, Zhou H, Gu L, Liu Y, Xu Q. SPP1, analyzed by bioinformatics methods, promotes the metastasis in colorectal cancer by activating EMT pathway. Biomed Pharmacother. 2017 Jul;91:1167-1177. doi: 10.1016/j.biopha.2017.05.056. Epub 2017 May 17. PMID: 28531945.

10.Song SZ, Lin S, Liu JN, Zhang MB, Du YT, Zhang DD, Xu WH, Wang HB. Targeting of SPP1 by microRNA-340 inhibits gastric cancer cell epithelial-mesenchymal transition through inhibition of the PI3K/AKT signaling pathway. J Cell Physiol. 2019 Aug;234(10):18587-18601. doi: 10.1002/jcp.28497. Epub 2019 Apr 5. PMID: 30953349.

11.Tsuchiya N, Sawada Y, Endo I, Saito K, Uemura Y, Nakatsura T. Biomarkers for the early diagnosis of hepatocellular carcinoma. World J Gastroenterol. 2015 Oct 7;21(37):10573-83. doi: 10.3748/wjg.v21.i37.10573. PMID: 26457017; PMCID: PMC4588079.

12. Liu M, Cao D, Russell R, Handschumacher RE, Pizzorno G. Expression, characterization, and detection of human uridine phosphorylase and identification of variant uridine phosphorolytic activity in selected human tumors. Cancer Res. 1998 Dec 1;58(23):5418-24. PMID: 9850074.

13. Wang J, Xu S, Lv W, Shi F, Mei S, Shan A, Xu J, Yang Y. Uridine phosphorylase 1 is a novel immune-related target and predicts worse survival in brain glioma. Cancer Med. 2020 Aug;9(16):5940-5947. doi: 10.1002/cam4.3251. Epub 2020 Jun 24. PMID: 32583596; PMCID: PMC7433823.
